# Deconvolving SARS-CoV-2 mRNA Vaccine Impact on Immunotherapy-Related Survival

**DOI:** 10.1158/2159-8290.CD-26-0403

**Published:** 2026-06-22

**Authors:** Justin Jee, Jiajia Zhang, Jessica A. Lavery, Michele Waters, Christoper J. Fong, Andy J. Minn, Michael S. Glickman, Katherine S. Panageas, Charles L. Sawyers, Nikolaus Schultz

**Affiliations:** 1 Memorial Sloan Kettering Cancer Center, New York, New York.; 2 Howard Hughes Medical Institute, Chevy Chase, Maryland.

## Abstract

Real-world data suggest that SARS-CoV-2 mRNA vaccines, administered within 100 days of immune checkpoint inhibitor (ICI) treatment (“peri-ICI vaccination”), may improve ICI effectiveness through synergistic immune priming. In an independent real-world cohort and reanalysis of a published cohort, both from tertiary cancer centers in the United States, multiple analyses did not support a treatment-synergy hypothesis. Applying a prior analytic framework, although longer survival with peri-ICI vaccination was observed at the start of the pandemic, this association was not seen in periods when vaccination was broadly available. Longer survival with vaccination in the early pandemic was also not specific to ICI therapies. Progression-free survival during periods of high vaccine uptake was not longer than in prevaccination periods. Together, these findings indicate that the previously reported vaccination survival advantage is largely explained by selection bias, with patients who had more favorable prognoses more likely to receive the SARS-CoV-2 vaccination, particularly in the early pandemic.

**Significance:**

The impact of tumor antigen–agnostic vaccination on immunotherapy response is unclear. In a reanalysis of published data and in an independent cohort, after adjustment for multiple confounders, SARS-CoV-2 mRNA vaccination did not improve immunotherapy-related survival in patients with cancer.

## Teaser

In analyses designed to control for potential confounders, SARS-CoV-2 mRNA vaccines did not seem to improve immunotherapy outcomes.

## Introduction

A recent study utilizing real-world data has suggested that SARS-CoV-2 mRNA vaccination is associated with improved overall survival (OS) among patients with cancer treated with immune checkpoint inhibitors (ICI; ref. 1). Preclinical studies further demonstrated that mRNA vaccination, independent of tumor neoantigen similarity, induces type I interferon signaling and improves CD8^+^ T-cell priming, providing a biologically plausible mechanism by which SARS-CoV-2 vaccination could augment ICI efficacy (1–3).

However, drawing reliable conclusions, whether associational or causal, from real-world survival data collected during an unprecedented global pandemic is challenging. Disruptions in cancer screening and treatment led to temporary shifts in the characteristics of patients receiving therapy (4). Population-level behavioral changes, including masking and quarantining (5), along with selection bias among those who chose to be vaccinated (6) collectively increase the potential for biased effect estimates. In addition, mRNA vaccines were instrumental in reducing the clinical severity and mortality of COVID-19 in a vulnerable cancer population (7–9), introducing another potential source of selection bias in analyses assessing vaccine-associated effects on cancer mortality in ICI-treated patients.

We leveraged a large, deeply annotated cohort (10) from a tertiary cancer center [Memorial Sloan Kettering Cancer Center (MSKCC)] and, when possible, reanalyzed published datasets (from MD Anderson Cancer Center) to evaluate the association between SARS-CoV-2 vaccination and survival following initiation of cancer treatment. To maximize ascertainment of vaccination status, we combined institutionally administered SARS-CoV-2 vaccination records, vaccination data captured outside our institution reported through health exchanges, clinic visit–associated patient surveys, and Artificial Intelligence (AI)-assisted natural language processing of free-text clinician notes. Using this dataset, we first evaluated the reproducibility of previously reported findings and confirmed that SARS-CoV-2 vaccination within 100 days of ICI initiation was associated with improved OS based on the previously published analytic approach (1). We then sought to determine whether:

1. The previously observed survival benefit was also present among patients who received SARS-CoV-2 vaccination more than 100 days prior to the start of treatment.
2. The association between SARS-CoV-2 vaccination and improved survival persisted during the later years of the pandemic (after 2021), when vaccination had become widespread.
3. The observed association was specific to patients receiving ICIs or was also present among patients treated with non-ICI antineoplastic therapies.
4. The survival benefit remained evident in landmark analyses restricted to periods during which all patients were eligible to receive peri-ICI vaccination.
5. Progression-free survival (PFS) was improved among patients with stage IV non–small cell lung cancer (NSCLC) treated during periods when SARS-CoV-2 vaccines were available.

## Results

We identified 8,346 patients treated with ICIs between 2017 and 2022 (characteristics shown in Supplementary Table S1) with a median follow-up of 3.4 (interquartile range, 2.4–5) years among patients alive at the end of the study. Among the 8,346 patients, 1,499 patients (18%) received SARS-CoV-2 vaccination within 100 days of ICI initiation (“peri-ICI vaccination”). In 2021 alone, 1,585 patients started ICI therapy, of whom 1,384 (87%) had received at least one vaccine dose. This high uptake was consistent with both local (11) and national (12) trends during this time period. Of 5,037 vaccine doses administered internally during 2020 to 2021, 4,998 (99%) were mRNA vaccines; 39 (1%) were non-mRNA vaccines.

Consistent with prior reports (1), the number of patients initiating ICI therapy declined during 2020 to 2021, likely a reflection of pandemic-related disruptions in healthcare delivery. It is noteworthy that most patients received the COVID-19 vaccination early in 2021, coinciding with the late 2020/early 2021 COVID-19 surge and initial vaccine availability (Supplementary Fig. S1). Of note, in 2021, patients receiving peri-ICI vaccination had better performance status than those who were not vaccinated, whereas no such difference was observed in 2022 (Supplementary Table S2).

Applying the previously published analytic specifications (1) to patients treated between 2017 and 2022, we similarly observed an association between peri-ICI vaccination and improved OS in multivariable Cox models adjusted for age, sex, stage, performance status, time since diagnosis, prior vaccination history, and cancer type, with comparable patterns observed within specific cancer types (Supplementary Fig. S2). Within this analytic framework, we also evaluated the relevance of the 100-day window used to define “peri-vaccination.” Notably, vaccination administered 100 to 200 days prior to ICI initiation as well as vaccination >200 days prior to ICI initiation were both similarly associated with longer OS compared with no vaccination (Supplementary Fig. S2).

**Figure 1.**
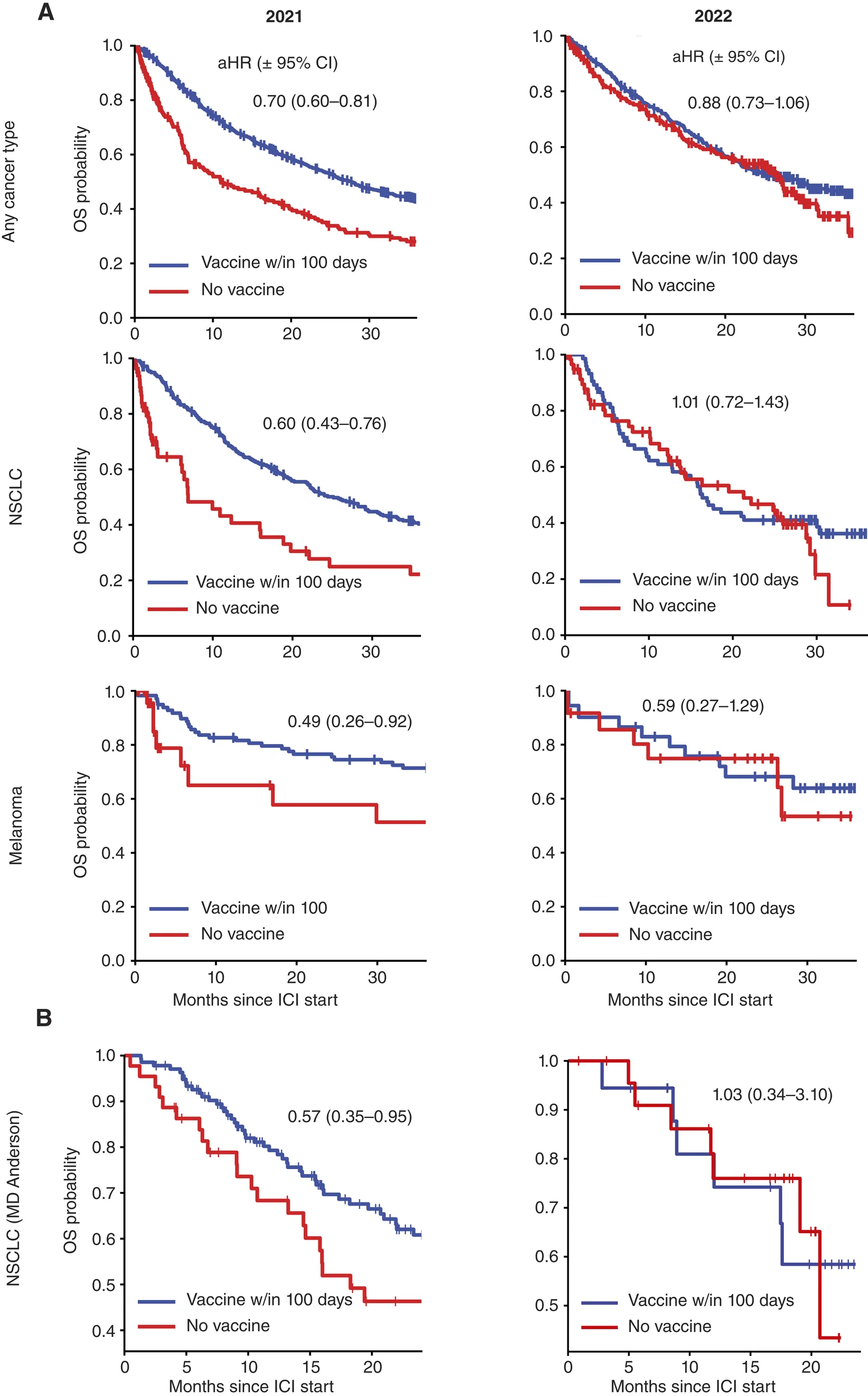
**A,** Survival by SARS-CoV-2 vaccination status following ICI start in 2021 and 2022. Vaccination within 100 days is treated as a time-dependent covariate to account for vaccinations given after ICI start. Analyses are risk-set–adjusted at the time of tumor genomic profiling. Adjusted HRs (aHR) for peri-ICI vaccination vs. no vaccination are adjusted for cancer type (for “any cancer type”), age, sex, stage, vaccination 100–200 days prior to treatment start, vaccination >200 days prior to treatment start, time since diagnosis, and performance status. Number of patients in each group and aHRs by cancer type are presented in Supplementary Fig. S3. **B,** OS following ICI start stratified by peri-ICI vaccination status and year in a previously published cohort of patients with NSCLC from MD Anderson Cancer Center. HRs are from univariable Cox proportional hazards models.

Cancer-directed therapies, including ICIs, have evolved over time, and multiple pandemic-era factors may have influenced the relationship between vaccination and ICI outcomes. Additionally, inclusion of data from 2017 to 2020, a prepandemic and largely prevaccine period during which patients initiating ICI therapy were, by definition, ineligible for peri-ICI vaccination, introduces additional potential for selection bias (13). We therefore examined the association between peri-ICI vaccination and OS separately by calendar year in 2021 and 2022. Peri-ICI vaccination was associated with longer OS among patients who initiated ICI therapy in 2021, but no such association was observed among those who started ICI in 2022 (Fig. 1A; Supplementary Fig. S3). To further corroborate this pattern, we reanalyzed the previously published cohort in which a survival benefit associated with peri-ICI vaccination had been reported, this time stratified by calendar year (1). Among patients with NSCLC treated at MD Anderson Cancer Center, peri-ICI vaccination was likewise not associated with longer OS among those who initiated therapy in 2022; although the sample sizes were limited, the hazard ratio (HR) was near null (Fig. 1B). Together, these results suggest that any potential survival advantage associated with peri-ICI vaccination diminished in time periods with fewer pandemic-era precautions (14).

**Figure 2.**
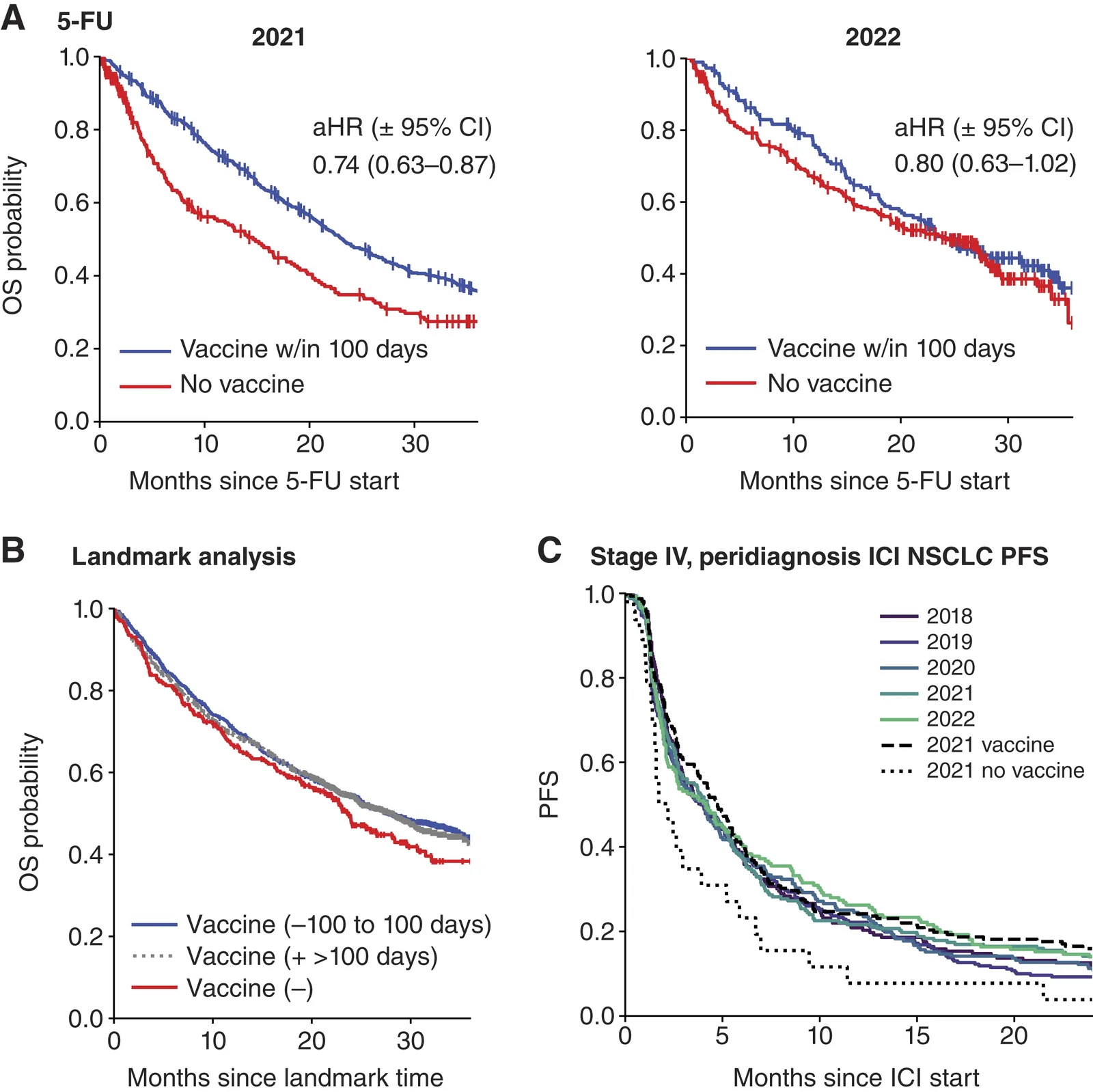
**A,** OS for patients treated with 5-FU in 2021 and 2022. Vaccination within 100 days is treated as a time-dependent covariate to account for vaccinations given after 5-FU start. Analyses are risk-set–adjusted at the time of tumor genomic profiling. Adjusted HRs (aHR) are adjusted for age, sex, cancer type, stage, vaccination 100–200 days prior to treatment start, vaccination >200 days prior to treatment start, time since diagnosis, and performance status. Number of patients and aHRs for the groups shown and specific cancer types are presented in Supplementary Fig. S4. Patients given 5-FU concurrently with immunotherapy were excluded from analysis. **B,** ICI-treated patients, including those treated from March 20, 2025, and landmarked at 100 days after ICI start to ensure equal eligibility for peri-ICI vaccination and prevent immortal time bias. **C,** PFS analysis including only patients with stage IV NSCLC given ICI vaccination within 180 days of diagnosis, stratified by year and, among patients starting ICI vaccination in 2021, stratified by whether they were ever vaccinated.

If peri-ICI vaccination improved outcomes through synergistic immune priming, any survival advantage would be expected to be restricted to patients receiving immune-based therapies. To test this hypothesis, we evaluated OS among patients treated with commonly administered anticancer agents that were not coadministered with ICIs. Among patients receiving 5-fluorouracil (5-FU), the most frequently used cytotoxic chemotherapy in our dataset, peri-treatment vaccination was associated with longer survival in 2021 but not in 2022, closely mirroring the temporal pattern observed among ICI-treated patients (Fig. 2A; Supplementary Fig. S4). Similarly, we observed a lack of treatment specificity in patients with advanced NSCLC treated with osimertinib, the most common tyrosine kinase inhibitor (TKI) in our dataset. In an analysis of patients treated from April 2018 through February 2022, i.e., when osimertinib was first approved for first-line treatment in NSCLC to the end of the omicron wave in New York, peri-TKI vaccination was associated with longer OS (Supplementary Fig. S5). No association between peri-TKI vaccination and survival was observed in 2021 or 2022, although the number of unvaccinated osimertinib-treated patients in 2021 was small (*n* = 73; Supplementary Fig. S5). Together, these findings suggest that the apparent survival advantage associated with SARS-CoV-2 vaccination is not specific to ICI treatment and therefore does not support an ICI-specific synergistic effect.

To address several potential biases not accounted for in the initial analyses, including population heterogeneity and changing treatment and vaccination practices over time in response to the COVID-19 pandemic, we performed landmark analyses at 100 days after ICI initiation and restricted the cohort to patients treated on or after March 20, 2021 (i.e., 100 days after vaccine availability). This approach fully captures peri-ICI vaccination exposure and ensures comparable follow-up among vaccinated and unvaccinated individuals. In these analyses, the observed association between vaccination (either peri-ICI vaccination or vaccination >100 days prior to ICI initiation) and improved survival dissipated (Fig. 2B). Notably, among patients starting ICI vaccination in 2021, the landmark approach excluded 21% of patients without peri-ICI vaccination but only 6% of patients with peri-ICI vaccination who died or were lost to follow-up prior to the landmark time point. The potential contribution of immortal time bias to the association between vaccination and survival was also noted in a landmark reanalysis of data from patients with NSCLC from MD Anderson Cancer Center. Although exact treatment start dates were not available for cohort restriction, peri-ICI vaccination from 2020 to 2022 was likewise no longer associated with improved survival benefit when analyses were landmarked at 100 days following ICI initiation (Supplementary Fig. S6).

If peri-ICI vaccination truly conferred benefit through a biological immune priming mechanism, outcomes should be superior in a population with a high SARS-CoV-2 mRNA vaccine uptake compared with a prevaccine era population. Evaluating this hypothesis using OS is challenging because survival may also be influenced by the increased use of ICIs in earlier stage disease and improvements in post-ICI lines of therapy over time, including the introduction of *KRAS* inhibitors (15). We therefore examined PFS in a more clinically homogenous cohort of patients with stage IV NSCLC who initiated ICI vaccination within 180 days of diagnosis, thereby reducing heterogeneity and limiting confounding from later-line treatments. In this cohort, PFS did not improve across calendar years, including during 2021 to 2022, when mRNA vaccine uptake was high, arguing against the hypothesis that vaccination improved disease control (Fig. 2C). Patients initiating ICI vaccination in 2021 who did not receive the mRNA vaccination demonstrated worse PFS than patients who were vaccinated (HR, 0.53; 95% CI, 0.36–0.79), suggesting that in 2021 vaccination was less common among patients with less favorable prognoses.

## Discussion

mRNA vaccination has proved to be a powerful tool for controlling infectious diseases (6, 16) and has generated substantial interest as a potential means of augmenting cancer immunotherapy (1, 17, 18). Preclinical studies provide a biologically plausible mechanism by which mRNA vaccination could synergize with ICI treatment in a tumor antigen–agnostic manner. However, estimating the true effect of SARS-CoV-2 mRNA vaccination on long-term cancer outcomes using observational real-world data is inherently challenging.

In our analysis, we replicated the previously reported association between peri-ICI vaccination and improved OS using a previously published analytic framework (1). Yet, multiple complementary analyses suggest that this apparent benefit was not specific to ICI therapy and was more likely driven by pandemic-era confounding and selection bias. First, the observed survival advantage extended to patients vaccinated more than 100 days before ICI initiation, a timeframe difficult to reconcile with a direct immune priming mechanism. Second, the benefit of vaccination was absent after 2021 in two independent datasets. Third, similar survival patterns were observed among patients receiving non-ICI therapies, including cytotoxic chemotherapy and TKIs. Finally, landmark analyses restricted to periods of broad vaccine availability, as well as PFS analyses in a clinically homogeneous cohort of patients with stage IV treatment-naïve NSCLC, failed to demonstrate a benefit associated with peri-ICI vaccination. Taken together, these results support the interpretation that vaccination status functioned primarily as a marker of favorable prognosis and engagement with the healthcare system during the pandemic, rather than as a mediator of enhanced antitumor immunity. Similar forms of bias have been described in prior vaccination studies (19) and are likely highly dependent on the care delivery context and patient behavior during the pandemic.

Recent prospective data underscore this distinction. A randomized trial demonstrated encouraging activity for the tumor antigen–targeting mRNA vaccine, imsapepimut plus etimupepimut in combination with pembrolizumab, in patients with melanoma (20). However, the magnitude of benefit observed with this targeted mRNA vaccine was substantially more modest than previously published real-world analyses of SARS-CoV-2 vaccination as a presumed immunopriming agent. If mRNA-based immune priming were a universal and robust mechanism for enhancing ICI efficacy, one would expect a neoantigen-directed mRNA vaccine to yield similarly strong, if not greater, improvements in ICI effectiveness. Instead, our findings suggest that at least part of the apparent survival advantage associated with SARS-CoV-2 vaccination reflects differences in healthcare access and utilization during the pandemic, including mitigation of delays in diagnosis, procedures, and treatment among vaccinated individuals.

Several limitations warrant consideration. This study was limited to two tertiary cancer centers in the United States. Associations between SARS-CoV-2 mRNA vaccination and immunotherapy outcomes may vary across regions because of differences in healthcare delivery practices, vaccine uptake, and shifts in infectious disease exposures before and after the COVID-19 lockdowns (5). Sample sizes for some cancer subtypes or for those treated with medications such as osimertinib were modest and should be interpreted cautiously. We restricted our analyses of peri-ICI vaccination to patients treated through 2022 because declining vaccination rates thereafter are difficult to disentangle from incomplete data capture and changing public health behavior. Additionally, fragmented healthcare records in the United States make it impossible to fully exclude the possibility of incomplete ascertainment of vaccination status.

Importantly, our findings should not be interpreted as evidence against the broader concept of immunopriming in cancer therapy. A growing body of evidence supports the potential for antigen-agnostic innate immune stimulation to improve ICI effectiveness, including mRNA-based approaches (1) such as the use of Bacillus Calmette–Guérin vaccines in bladder cancer (21) and Toll-like receptor 7/8 agonists in NSCLC (22). Other factors such as the microbiome (23) and direct infection exposure (24) may also incite antitumor immunity. However, our results indicate that the observed pandemic-era association between peri-ICI SARS-CoV-2 vaccination and improved survival is more plausibly explained by selection bias and other pandemic-related confounding factors than by a robust antigen-agnostic mRNA vaccine effect. These findings highlight the need for caution when interpreting observational datasets generated during an unprecedented pandemic.

## Methods

### Patients

We studied patients treated at MSKCC, a tertiary cancer center in New York. The study included patients from a pan-cancer, updated instance of the MSK Clinicogenomic Harmonized Oncologic Real-world Dataset (MSK-CHORD) cohort, including all patients with tumor genomic profiling with MSK-IMPACT or MSK-ACCESS, our institutional sequencing assays (10). Patients were included if they initiated treatment with ICIs, 5-FU, or osimertinib (advanced NSCLC only) between January 1, 2017, and December 31, 2022. Vital status was last ascertained on February 1, 2025. To reduce confounding by use of treatments in atypical cancer types, for ICI and 5-FU analyses, only cancer types with at least 500 patients treated with that respective medication were included. Patients receiving concurrent 5-FU and ICIs were only included in the ICI analyses. The study was approved by the MSKCC Institutional Review Board, which waived the requirement for informed consent given the retrospective nature of the analysis. For reanalyses of published data, OS data from the MD Anderson NSCLC cohort [Grippin and colleagues (1); Supplementary Fig. S1] were used.

### Data Annotation

Treatment, performance status, radiographic progression, diagnosis date, stage, and cancer type were annotated using previously described methods (10). For PFS analyses, an event was defined as the first radiographic progression after ICI initiation or death, whichever was first. To decrease the possibility that radiographic progression seen shortly after ICI initiation was not treatment-related, only radiographic progression after 30 days was considered; deaths at any time were considered events. Patients were right-censored at the date of the last follow-up. Receipt of concurrent chemotherapy was annotated based on whether chemotherapy was administered on the same start date as ICI therapy. SARS-CoV-2 vaccination data, including dates, were obtained from a combination of structured surveys, institutional testing and administration records, nursing survey data, health exchange data from vaccination data outside MSKCC, and free-text reports analyzed by AI large language models (LLM; Claude Sonnet 4.5). Clinician review (by J. Zhang and J. Jee) of 40 randomly selected vaccination cases found three cases in which vaccination status may be a false negative and 40 negative cases, none of which were deemed positive by the model. In all three false-negative cases, there was disparate documentation across an individual patient’s health record regarding vaccination status. All dates in the manually reviewed subset were abstracted correctly by the LLM.

### Statistical Analysis

No statistical method was used to predetermine the sample size in this observational study. Peri-ICI vaccination status was defined as the receipt of a SARS-CoV-2 mRNA vaccination within 100 days before or after treatment initiation. For patients with multiple vaccinations within the window, the vaccination closest to treatment initiation was selected.

OS by vaccination status was estimated using Cox proportional hazards models. Patients alive at the end of the study period were censored at their last visit date. Because tumor genomic profiling with MSK-IMPACT was an inclusion criterion for MSK-CHORD, all analyses were risk-set–adjusted at the time of first tumor sequencing (25). When applying the previously published analytic approach, OS was estimated from treatment initiation and estimated using multivariable Cox proportional hazards models, fitted overall and by cancer subtypes, including peri-ICI vaccination status, vaccination 100 to 200 days prior to treatment start, vaccination >200 days prior to treatment start, disease stage, performance (Eastern Cooperative Oncology Group, as a continuous variable), and time since diagnosis. Pan-cancer analyses were also adjusted for cancer type. Peri-ICI vaccination was treated as a time-dependent covariate to account for patients receiving peri-ICI vaccination after ICI start (patients vaccinated more than 100 days after treatment initiation remained in the no peri-ICI group). Patients vaccinated within the 100 days prior to treatment initiation were in the peri-ICI group at baseline. Survival was visualized using the Simon–Makuch method to facilitate the visualization of a time-dependent covariate (26). Fewer than 8% of patients in each treatment cohort had missing data in at least one variable (either time since diagnosis or performance status) and were excluded from the multivariable analysis. Secondary analyses using the previously published framework on our institutional data, and when possible, the MD Anderson data, were performed: (i) with a broader definition of peri-ICI vaccination, in which vaccination 100 to 200 days and >200 days prior to ICI initiation were additionally evaluated; (ii) stratified by year, among patients beginning ICI in 2021 or 2022; (iii) among patients treated with other commonly administered anticancer agents (5-FU and osimertinib) not coadministered with ICI therapy.

To address potential immortal time and selection bias related to vaccine administration, analyses evaluating peri-ICI vaccination and vaccination >100 days prior to ICI initiation were also performed in our institutional data and in the original MD Anderson data using a 100-day landmark after treatment initiation. In our institutional data, this analysis was restricted to patients whose treatment started March 20, 2021, onward, reflecting the period in which vaccine availability was widespread; in the analysis of the MD Anderson data, analyses were restricted to 2020 to 2022 because the exact treatment start dates were not available. Patients who died or were lost to follow-up prior to 100 days following treatment initiation were excluded from these analyses.

Among MSKCC patients with stage IV NSCLC initiating treatment within 180 days of diagnosis, PFS by year and vaccination status (in 2021) was estimated using Kaplan–Meier methods with risk-set adjustment for left truncation, treating peri-vaccination status as a time-dependent covariate. Analysis included patients initiating treatment from 2018 onward, when ICI became a standard part of first-line care for patients with driver-negative NSCLC. Patients who progressed prior to genomic sequencing were excluded from the risk set at the time of regimen initiation. A sensitivity analysis was performed without risk-set adjustment for left truncation to include patients who progressed prior to sequencing.

Analyses were performed in Python 3.10.11 (RRID: SCR_008394) using lifelines 0.26.0. The study was performed according to Strengthening the Reporting of Observational Studies in Epidemiology (STROBE) guidelines (Supplementary Information: STROBE Checklist S1). All code and line-level data are available at https://github.com/clinical-data-mining/covid_vaccine_io.

## Supplementary Material

Supplementary Tables S1-S2 Table S1. Characteristics of patients treated with immune checkpoint inhibitors (ICI). Table S2. Characteristics of patients treated with immune checkpoint inhibitors (ICI) stratified by year of ICI start.

Figure S1 Figure S1. Changing event rates over time.

Figure S2 Figure S2. Survival following ICI start stratified by SARS-CoV-2 vaccination status from 2017-2022.

Figure S3 Figure S3. Subgroup analysis for ICI-treated patients.

Figure S4 Figure S4. Subgroup analyses for 5-FU-treated patients

Figure S5 Figure S5. Overall survival for patients treated with osimertinib.

Figure S6 Figure S6. Landmarked analysis in the MD Anderson cohort (NSCLC) starting ICI 2020-2022.

STROBE checklist 1 STROBE checklist

## Data Availability

All data to replicate the analyses in this study are available in a Zenodo (RRID: SCR_004129) release at https://doi.org/10.5281/zenodo.21896000.

## References

[1] Grippin AJ , Marconi C, Copling S, Li N, Braun C, Woody C, . SARS-CoV-2 mRNA vaccines sensitize tumours to immune checkpoint blockade. Nature 2025;647:488–97. 10.1038/s41586-025-09655-y. PMC12611756 41125896

[2] Li C , Lee A, Grigoryan L, Arunachalam PS, Scott MK, Trisal M, . Mechanisms of innate and adaptive immunity to the Pfizer-BioNTech BNT162b2 vaccine. Nat Immunol 2022;23:543–55. 10.1038/s41590-022-01163-9. PMC8989677 35288714

[3] Qdaisat S , Wummer B, Stover BD, Zhang D, McGuiness J, Weidert F, . Sensitization of tumours to immunotherapy by boosting early type-I interferon responses enables epitope spreading. Nat Biomed Eng 2025;9:1437–52. 10.1038/s41551-025-01380-1. 40681861

[4] Richards M , Anderson M, Carter P, Ebert BL, Mossialos E. The impact of the COVID-19 pandemic on cancer care. Nat Cancer 2020;1:565–7. 10.1038/s43018-020-0074-y. PMC7238956 35121972

[5] Brett TS , Rohani P. Collateral effects of COVID-19 pandemic control on the US infectious disease landscape. Science 2025;390:510. 10.1126/science.adw4964. 41166479

[6] Cai M , Xie Y, Al-Aly Z. Association of 2024–2025 Covid-19 vaccine with Covid-19 outcomes in U.S. veterans. N Engl J Med 2025;393:1612–23. 10.1056/NEJMoa2510226. 41061231

[7] Jee J , Foote MB, Lumish M, Stonestrom AJ, Wills B, Narendra V, . Chemotherapy and COVID-19 outcomes in patients with cancer. J Clin Oncol 2020;38:3538–46. 10.1200/JCO.20.01307. PMC7571792 32795225

[8] Kirkwood MK , Aggarwal C, Bruinooge SS, Williams JH, Kaltenbaugh M, Ramos N, . Differential survival among patients with cancer by COVID-19 vaccination status: an analysis of the ASCO COVID-19 registry. JCO Oncol Adv 2024;1:e2400034. 10.1200/oa-24-00034.

[9] Skarbinski J , Elkin EP, Ziemba YC, Kazemian E, Wilson BM, Siddiqui H, . COVID-19 vaccine booster uptake and effectiveness among US adults with cancer. JAMA Oncol 2025;11:999–1010. 10.1001/jamaoncol.2025.2020. PMC12272354 40674059

[10] Jee J , Fong C, Pichotta K, Tran TN, Luthra A, Waters M, . Automated real-world data integration improves cancer outcome prediction. Nature 2024;636:728–36. 10.1038/s41586-024-08167-5. PMC11655358 39506116

[11] Sah P , Vilches TN, Moghadas SM, Pandey A, Gondi S, Schneider EC, . Return on investment of the COVID-19 vaccination campaign in New York City. JAMA Netw Open 2022;5:e2243127. 10.1001/jamanetworkopen.2022.43127. PMC9679875 36409495

[12] Nguyen KH , Zhao R, Chen S, Bednarczyk RA. Exclusive and dual influenza and COVID-19 vaccination among U.S. adults and adolescents in 2021. Ann Med 2023;55:2196436. 10.1080/07853890.2023.2196436. PMC10116913 37052255

[13] Schemper M , Smith TL. A note on quantifying follow-up in studies of failure time. Control Clin Trials 1996;17:343–6. 10.1016/0197-2456(96)00075-x. 8889347

[14] Treisman R . New York lifts indoor mask mandate, with California and N.J. mandates also set to end. NPR. 2022 [cited 2026 Jun 12]. Available from: https://www.npr.org/2022/02/09/1079464501/new-york-mask-mandate-california-new-jersey.

[15] Skoulidis F , Li BT, Dy GK, Price TJ, Falchook GS, Wolf J, . Sotorasib for lung cancers with KRAS p.G12C mutation. N Engl J Med 2021;384:2371–81. 10.1056/NEJMoa2103695. PMC9116274 34096690

[16] Polack FP , Thomas SJ, Kitchin N, Absalon J, Gurtman A, Lockhart S, . Safety and efficacy of the BNT162b2 mRNA Covid-19 vaccine. N Engl J Med 2020;383:2603–15. 10.1056/NEJMoa2034577. PMC7745181 33301246

[17] Wang JD , Puglisi L, Bagsic SR, Bollin KB, New J. Association of co-administration of a vaccine with immune checkpoint inhibitors with survival. J Clin Oncol 2025;43(16_suppl):11151. 10.1200/JCO.2025.43.16_suppl.11151.

[18] Lorentzen CL , Haanen JB, Met Ö, Svane IM. Clinical advances and ongoing trials on mRNA vaccines for cancer treatment. Lancet Oncol 2022;23:e450–8. 10.1016/S1470-2045(22)00372-2. PMC9512276 36174631

[19] Eurich DT , Marrie TJ, Johnstone J, Majumdar SR. Mortality reduction with influenza vaccine in patients with pneumonia outside “flu” season: pleiotropic benefits or residual confounding? Am J Respir Crit Care Med 2008;178:527–33. 10.1164/rccm.200802-282OC. 18556629

[20] Hassel JC , Arance AM, Carlino MS, Ascierto PA, Sandhu SK, Puzanov I, . LBA53 IO102-IO103 cancer vaccine plus pembrolizumab for first-line (1L) advanced melanoma: primary phase III results (IOB-013/KN-D18). Ann Oncol 2025;36(suppl 2):S1712–13. 10.1016/j.annonc.2025.09.065.

[21] Daman AW , Antonelli AC, Redelman-Sidi G, Paddock L, Khayat S, Ketavarapu M, . Microbial cancer immunotherapy reprograms hematopoiesis to enhance myeloid-driven anti-tumor immunity. Cancer Cell 2025;43:1442–59.e10. 10.1016/j.ccell.2025.05.002. PMC12377364 40446799

[22] Costin D , Jotte R, Kukunoor RN, Al-Jazayrly GM, Goldsschmidt J, Rybstein M, . 1850MO TeLuRide-005: phase II study of EIK1001, a toll-like receptor 7/8 (TLR7/8) co-agonist with pembrolizumab (pembro) + chemotherapy (chemo) as first-line (1L) therapy in stage 4 non-small cell lung cancer (NSCLC). Ann Oncol 2025;36(suppl 2):S995. 10.1016/j.annonc.2025.08.2474.

[23] Elkrief A , Pidgeon R, Maleki Vareki S, Messaoudene M, Castagner B, Routy B. The gut microbiome as a target in cancer immunotherapy: opportunities and challenges for drug development. Nat Rev Drug Discov 2025;24:685–704. 10.1038/s41573-025-01211-7. 40457025

[24] Meo C , Palma G, Bruzzese F, Budillon A, Napoli C, de Nigris F. Spontaneous cancer remission after COVID-19: insights from the pandemic and their relevance for cancer treatment. J Transl Med 2023;21:273. 10.1186/s12967-023-04110-w. PMC10119533 37085802

[25] Brown S , Lavery JA, Shen R, Martin AS, Kehl KL, Sweeney SM, . Implications of selection bias due to delayed study entry in clinical genomic studies. JAMA Oncol 2022;8:287–91. 10.1001/jamaoncol.2021.5153. PMC9190030 34734967

[26] Simon R , Makuch RW. A non-parametric graphical representation of the relationship between survival and the occurrence of an event: application to responder versus non-responder bias. Stat Med 1984;3:35–44. 10.1002/sim.4780030106. 6729287

