# Supplementary material for: Deconvolving SARS-CoV-2 mRNA Vaccine Impact on Immunotherapy-Related Survival
Source: Cancer Discov. 2026 Jun 22;16(11):2235–41. doi: 10.1158/2159-8290.CD-26-0403 (PMC13725333; doi:10.1158/2159-8290.CD-26-0403)
Supplement: Supplementary file 1 [file cd-26-0403_figure_s1_suppsf1.pdf]

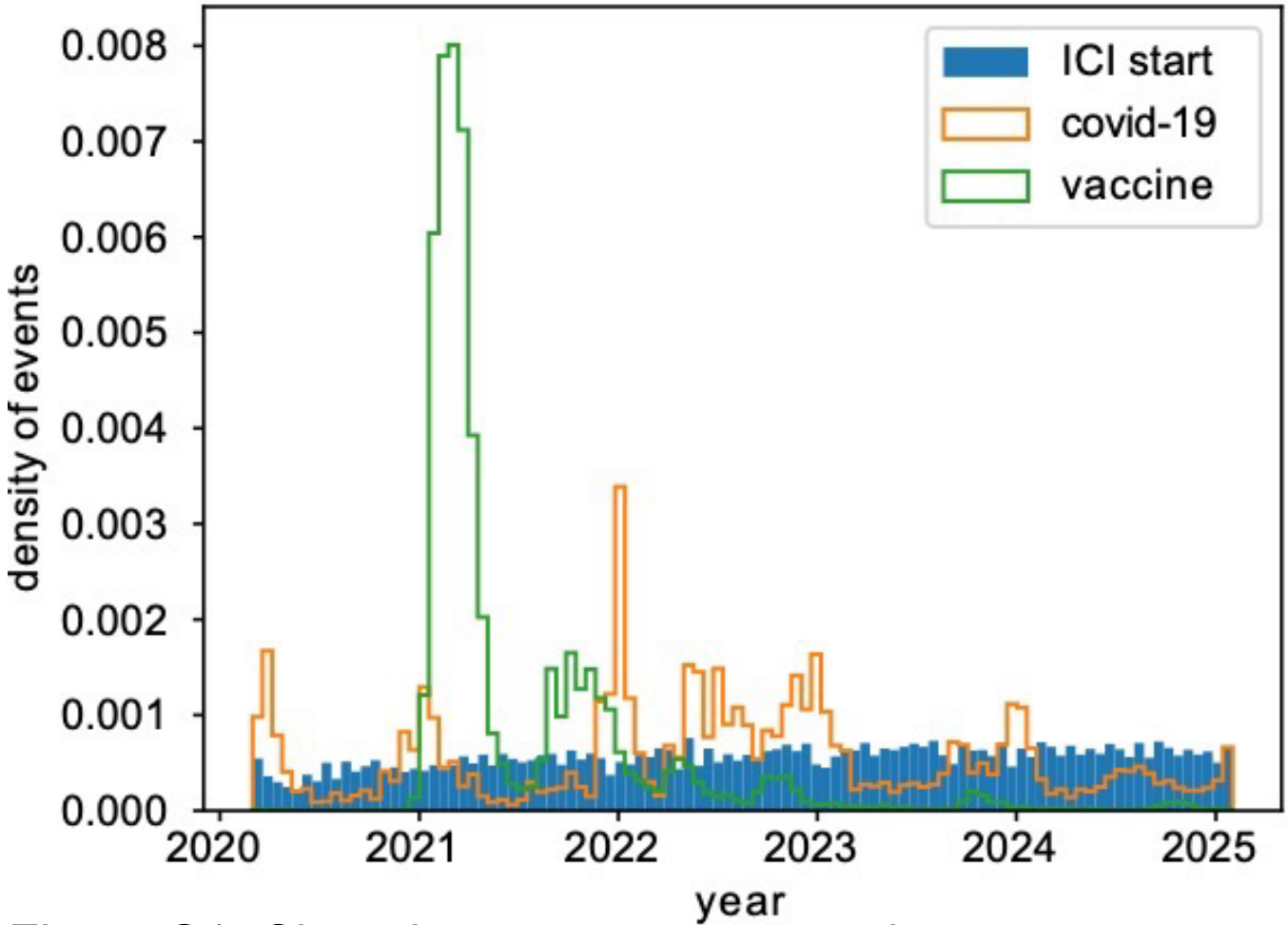

Figure S1. Changing event rates over time.
SARS-CoV-2 infection and vaccination rates, as well as immune checkpoint inhibitor (ICI) administration over time
