# Supplementary material for: Deconvolving SARS-CoV-2 mRNA Vaccine Impact on Immunotherapy-Related Survival
Source: Cancer Discov. 2026 Jun 22;16(11):2235–41. doi: 10.1158/2159-8290.CD-26-0403 (PMC13725333; doi:10.1158/2159-8290.CD-26-0403)
Supplement: Supplementary file 2 [file cd-26-0403_figure_s2_suppsf2.pdf]

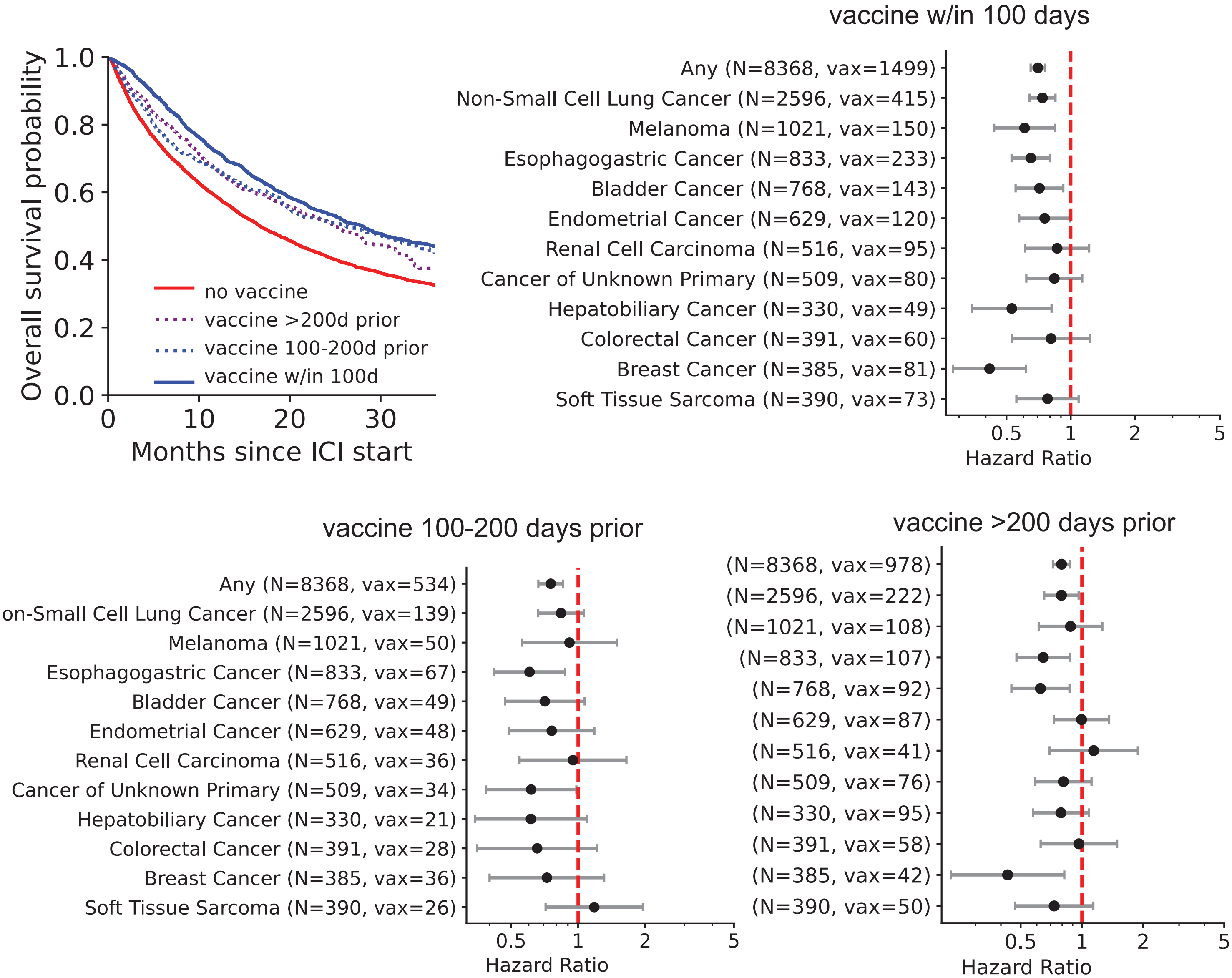

Figure S2. Survival following ICI start stratified by SARS-CoV-2 vaccination status from 2017-2022. Kaplan-Meier overall survival curve for is shown, with peri-ICI vaccination after ICI start treated as a time-dependent covariate and risk set adjusted at time of tumor genomic profiling. Forest plots show adjusted Cox proportional hazard ratios +/- 95%CI for peri-ICI vaccination, 100-200 days before ICI start, and >200 days before ICI start vs those without peri-ICI vaccination, stratified by cancer type ("vax" = vaccinated group according to the figure heading). Hazard ratios are adjusted for age, sex (except for breast and endometrial cancer models), disease stage, ECOG, time since diagnosis, and vaccination either within 100 days, 100-200 days prior, or >200 days prior to ICI initiation.
