# Supplementary material for: Deconvolving SARS-CoV-2 mRNA Vaccine Impact on Immunotherapy-Related Survival
Source: Cancer Discov. 2026 Jun 22;16(11):2235–41. doi: 10.1158/2159-8290.CD-26-0403 (PMC13725333; doi:10.1158/2159-8290.CD-26-0403)
Supplement: Supplementary file 4 [file cd-26-0403_figure_s4_suppsf4.pdf]

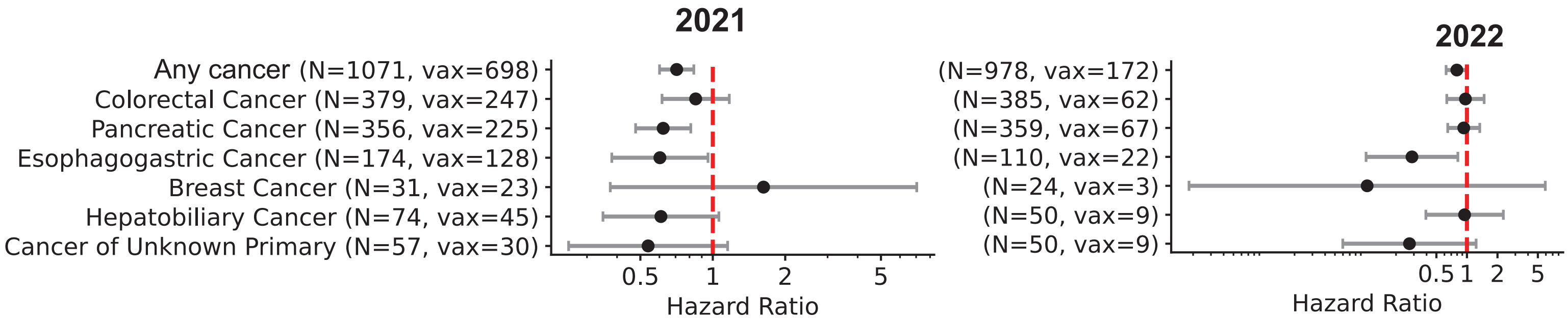


Figure S4. Subgroup analyses for 5-FU-treated patients

Forest plots show hazard ratios for peri-5-FU vaccination (“vax”) and are adjusted for age, sex (except for breast cancer model), disease stage, ECOG, prior vaccination, vaccination 100-200 days prior to treatment start, vaccination >200 days prior to treatment start, and time since diagnosis. The pan-cancer hazard ratiois also adjusted for cancer type. Patients treated with simultaneous ICI are excluded. Analyses are risk set adjusted at time of tumor genomic profiling.
