# Supplementary material for: Deconvolving SARS-CoV-2 mRNA Vaccine Impact on Immunotherapy-Related Survival
Source: Cancer Discov. 2026 Jun 22;16(11):2235–41. doi: 10.1158/2159-8290.CD-26-0403 (PMC13725333; doi:10.1158/2159-8290.CD-26-0403)
Supplement: Supplementary file 5 [file cd-26-0403_figure_s5_suppsf5.pdf]

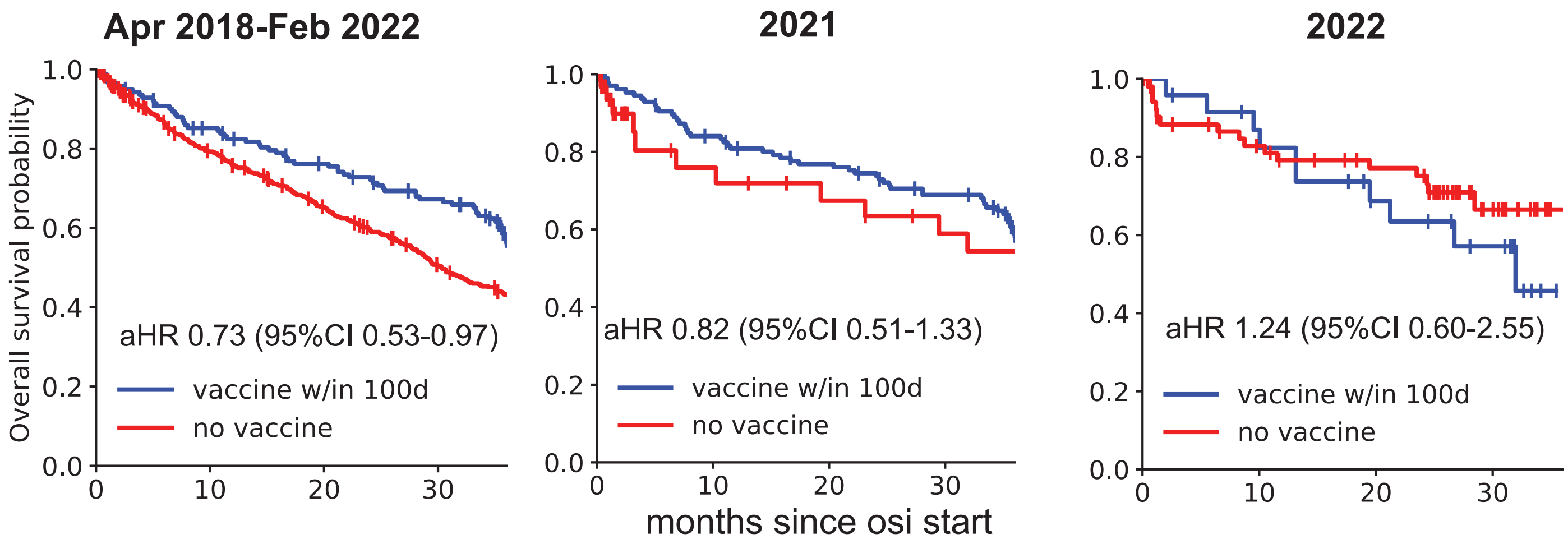


Figure S5. Overall survival for patients treated with osimertinib.
Subanalyses performed by the years shown. Vaccination within 100 days is treated as a time-dependent covariate to account for vaccinations given after treatment start. Analyses are risk-set adjusted at time of risk set entry (tumor genomic profiling). Hazard ratios are adjusted for age, sex, time since diagnosis, vaccination 100-200 days prior to treatment start, vaccination >200 days prior to treatment start, and performance status.
