# Supplementary material for: Deconvolving SARS-CoV-2 mRNA Vaccine Impact on Immunotherapy-Related Survival
Source: Cancer Discov. 2026 Jun 22;16(11):2235–41. doi: 10.1158/2159-8290.CD-26-0403 (PMC13725333; doi:10.1158/2159-8290.CD-26-0403)
Supplement: Supplementary file 6 [file cd-26-0403_figure_s6_suppsf6.pdf]

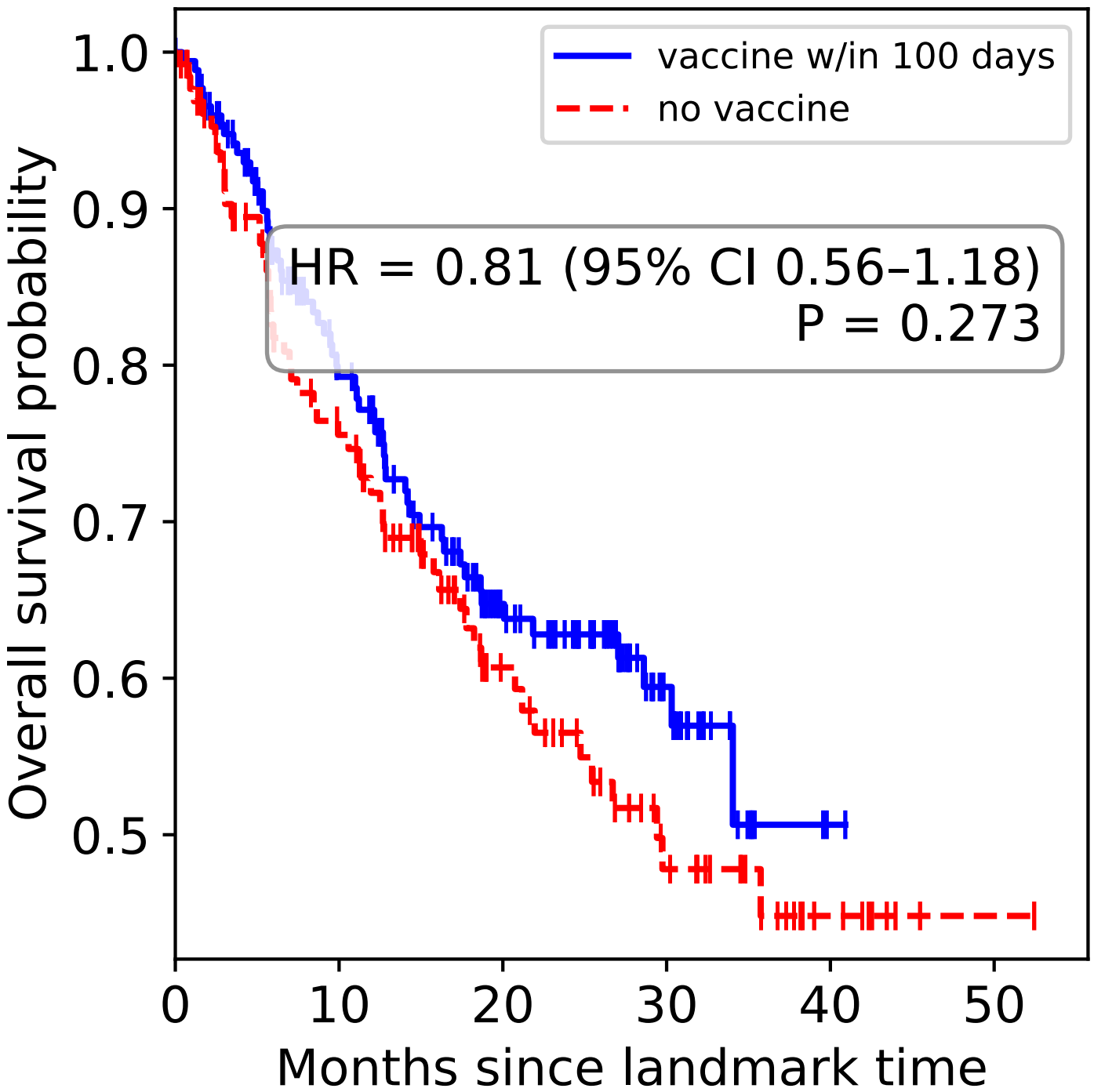

vaccine w/in 100 days						
At risk	175	114	68	24	1	0
no vaccine						
At risk	130	84	44	24	8	1

Figure S6. Landmarked analysis in the MD Anderson cohort (NSCLC) starting ICI 2020-2022. Landmarking is done at 100 days following ICI initiation to prevent immortal time bias. HR is estimated from a univariable Cox proportional hazards model.
