# Supplementary material for: Deconvolving SARS-CoV-2 mRNA Vaccine Impact on Immunotherapy-Related Survival
Source: Cancer Discov. 2026 Jun 22;16(11):2235–41. doi: 10.1158/2159-8290.CD-26-0403 (PMC13725333; doi:10.1158/2159-8290.CD-26-0403)
Supplement: Supplementary file 7 [file cd-26-0403_strobe_checklist_1_suppsd1.docx]

STROBE Statement—checklist of items that should be included in reports of observational studies

|  | Item No. | Recommendation | Page  No. | Relevant text from manuscript |
| --- | --- | --- | --- | --- |
| **Title and abstract** | 1 | (*a*) Indicate the study’s design with a commonly used term in the title or the abstract | 2 | Abstract describes analysis of real-world data, an independent dataset (MSK-CHORD), and re-analysis of a published cohort. |
|  |  | (*b*) Provide in the abstract an informative and balanced summary of what was done and what was found | 2 | The abstract states the exposure, datasets, comparative analyses, and principal finding that the observed survival association likely reflects selection bias rather than vaccine-mediated immune priming. |
| Introduction | | | |  |
| Background/rationale | 2 | Explain the scientific background and rationale for the investigation being reported | 3 | The introduction reviews prior real-world and preclinical evidence for vaccine-related immune priming, then explains why pandemic-era confounding, behavior change, and selection bias require re-evaluation. |
| Objectives | 3 | State specific objectives, including any prespecified hypotheses | 3-4 | Specific objectives are listed explicitly: whether benefit extends beyond 100 days, persists after 2021, is limited to ICI, remains after landmarking to vaccine-eligible periods, and is seen in PFS among treatment-naive stage IV NSCLC. |
| Methods | | | |  |
| Study design | 4 | Present key elements of study design early in the paper | 3; 9-10 | Early text describes a large real-world MSK cohort with re-analysis of published MD Anderson data; Methods later clarify the observational cohort-style survival analyses performed on institutional and external datasets. |
| Setting | 5 | Describe the setting, locations, and relevant dates, including periods of recruitment, exposure, follow-up, and data collection | 9-10 | Setting is Memorial Sloan Kettering Cancer Center in New York, using the pan-cancer MSK-CHORD cohort. Treatment initiation spanned Jan 1, 2017 to Dec 31, 2022, with vital status last ascertained Feb 1, 2025; an external MD Anderson NSCLC cohort was also re-analyzed. |
| Participants | 6 | (*a*) *Cohort study*—Give the eligibility criteria, and the sources and methods of selection of participants. Describe methods of follow-up  *Case-control study*—Give the eligibility criteria, and the sources and methods of case ascertainment and control selection. Give the rationale for the choice of cases and controls  *Cross-sectional study*—Give the eligibility criteria, and the sources and methods of selection of participants | 9-10 | Eligibility criteria and selection are described for patients initiating ICI, 5-FU, or osimertinib (NSCLC only). Common-cancer restrictions, handling of concurrent 5-FU plus ICI, external MD Anderson data use, censoring, and landmark exclusions are reported. |
|  |  | (*b*) *Cohort study*—For matched studies, give matching criteria and number of exposed and unexposed  *Case-control study*—For matched studies, give matching criteria and the number of controls per case | NA | Not a matched cohort study. |
| Variables | 7 | Clearly define all outcomes, exposures, predictors, potential confounders, and effect modifiers. Give diagnostic criteria, if applicable | 9-10 | The manuscript defines the exposure (peri-ICI vaccination within 100 days before or after treatment), outcomes (overall survival and progression-free survival), and covariates/confounders including age, sex, stage, ECOG performance status, time since diagnosis, and cancer type. |
| Data sources/ measurement | 8* | For each variable of interest, give sources of data and details of methods of assessment (measurement). Describe comparability of assessment methods if there is more than one group | 9-10 | Clinical variables were annotated using previously described methods. Vaccination data were assembled from survey data, institutional records, nursing survey data, health-exchange data, and LLM-assisted abstraction from free text with clinician review of a random sample. |
| Bias | 9 | Describe any efforts to address potential sources of bias | 10 | Bias mitigation included risk-set adjustment at first tumor sequencing, use of time-dependent vaccination status when vaccination occurred after treatment start, and 100-day landmark analyses restricted to vaccine-eligible periods. |
| Study size | 10 | Explain how the study size was arrived at | 9 | No formal sample-size calculation is reported. The cohorts appear to include all eligible patients in the available dataset during 2017-2022, with additional restriction to cancer types with at least 500 treated patients for ICI and 5-FU analyses. |

Continued on next page

| Quantitative variables | 11 | Explain how quantitative variables were handled in the analyses. If applicable, describe which groupings were chosen and why | 10 | Quantitative handling includes the 100-day peri-vaccination window, additional 100-200 day and >200 day categories, calendar-year stratification (2021 vs 2022), and restriction to NSCLC patients initiating treatment within 90 days of diagnosis for PFS analyses. |
| --- | --- | --- | --- | --- |
| Statistical methods | 12 | (*a*) Describe all statistical methods, including those used to control for confounding | 10-11 | Overall survival and PFS were analyzed using Kaplan-Meier methods and Cox proportional hazards models. Multivariable models adjusted for disease stage, ECOG, time since diagnosis, vaccination status, and cancer type as applicable. |
|  |  | (*b*) Describe any methods used to examine subgroups and interactions | 10 | Subgroup or stratified analyses were performed overall and by cancer type, by calendar year, by treatment class (ICI, 5-FU, osimertinib), by broader vaccination timing categories, and in treatment-naive stage IV NSCLC. |
|  |  | (*c*) Explain how missing data were addressed | 10 | Fewer than 8% of patients in each treatment cohort had missing data in at least one covariate (time since diagnosis or performance status) and were excluded from multivariable analysis. |
|  |  | (*d*) *Cohort study*—If applicable, explain how loss to follow-up was addressed  *Case-control study*—If applicable, explain how matching of cases and controls was addressed  *Cross-sectional study*—If applicable, describe analytical methods taking account of sampling strategy | 9-10 | Patients were censored at last visit date for OS and at last follow-up for PFS. For landmark analyses, patients who died or were lost to follow-up before 100 days after treatment initiation were excluded. |
|  |  | (*e*) Describe any sensitivity analyses | 10 | Secondary or sensitivity analyses included broader vaccination windows, year-stratified analyses, non-ICI treatment analyses, 100-day landmark analyses, and NSCLC PFS analyses. |
| Results | | | | |
| Participants | 13* | (a) Report numbers of individuals at each stage of study—eg numbers potentially eligible, examined for eligibility, confirmed eligible, included in the study, completing follow-up, and analysed | 4; 24-26 | The manuscript reports numbers in the main analytic groups, such as 8,368 ICI-treated patients and 1,410 peri-ICI-vaccinated patients, with subgroup counts in Tables S1 and S2. |
|  |  | (b) Give reasons for non-participation at each stage | 9-10 | Some exclusions are stated, including rare cancer types, handling of concurrent 5-FU plus ICI, and exclusion of patients who died or were lost to follow-up before the landmark time. |
|  |  | (c) Consider use of a flow diagram | NA | NA |
| Descriptive data | 14* | (a) Give characteristics of study participants (eg demographic, clinical, social) and information on exposures and potential confounders | 24-26 | Tables S1 and S2 report participant characteristics including cancer type, concurrent cytotoxic therapy, age, sex, stage, ECOG performance status, and time from diagnosis to treatment, overall and by vaccination group or year. |
|  |  | (b) Indicate number of participants with missing data for each variable of interest | 10 | Missingness is reported in the Methods section and in Table S1 |
|  |  | (c) *Cohort study*—Summarise follow-up time (eg, average and total amount) | 9-10 | First sentence of Results describes the median follow up for patients in the cohort. |
| Outcome data | 15* | *Cohort study*—Report numbers of outcome events or summary measures over time | 4-6; 16-23 | Outcome data are reported through Kaplan-Meier curves and hazard ratios for overall survival and progression-free survival across overall, year-stratified, external-cohort, non-ICI, landmark, and NSCLC analyses. |
|  |  | *Case-control study—*Report numbers in each exposure category, or summary measures of exposure | NA | Not a case-control study. |
|  |  | *Cross-sectional study—*Report numbers of outcome events or summary measures | NA | Not a cross-sectional study. |
| Main results | 16 | (*a*) Give unadjusted estimates and, if applicable, confounder-adjusted estimates and their precision (eg, 95% confidence interval). Make clear which confounders were adjusted for and why they were included | 4-6; 10; 16-23 | The manuscript reports adjusted hazard ratios with confidence intervals in figures and forest plots, and specifies the confounders used for adjustment, including age, sex, stage, ECOG, time since diagnosis, and cancer type as applicable. |
|  |  | (*b*) Report category boundaries when continuous variables were categorized | 10 | Category boundaries are reported for vaccination timing (within 100 days, 100-200 days, and >200 days before treatment), calendar year (2021 and 2022), and NSCLC PFS eligibility (treatment within 90 days of diagnosis). |
|  |  | (*c*) If relevant, consider translating estimates of relative risk into absolute risk for a meaningful time period | NR | Hazard ratios and survival curves are reported, but the paper does not translate relative effects into absolute risk differences over a defined time period. |

Continued on next page

| Other analyses | 17 | Report other analyses done—eg analyses of subgroups and interactions, and sensitivity analyses | 5-6; 10; 17-23 | Other analyses include broader vaccination timing categories, year-specific analyses, re-analysis of the MD Anderson cohort, non-ICI therapy analyses, 100-day landmark analyses, and treatment-naive NSCLC PFS analyses. |
| --- | --- | --- | --- | --- |
| Discussion | | | | |
| Key results | 18 | Summarise key results with reference to study objectives | 2; 6-8 | The abstract and discussion summarize the main findings: the previously reported association is reproduced under the prior framework, but it is not ICI-specific, fades after 2021, disappears in landmark or PFS analyses, and is most consistent with selection bias. |
| Limitations | 19 | Discuss limitations of the study, taking into account sources of potential bias or imprecision. Discuss both direction and magnitude of any potential bias | 7 | Limitations discussed include the single-center urban setting, small subgroup sizes, possible residual or unmeasured confounding, incomplete vaccine capture, pandemic-related practice changes, and lack of adjustment for subsequent therapies. |
| Interpretation | 20 | Give a cautious overall interpretation of results considering objectives, limitations, multiplicity of analyses, results from similar studies, and other relevant evidence | 6-8 | The interpretation is cautious and integrates the study objectives, multiple analyses, limitations, prior preclinical and clinical evidence, and the possibility that pandemic-era observational associations do not reflect a biologic immune-priming effect. |
| Generalisability | 21 | Discuss the generalisability (external validity) of the study results | 7-8 | Generalisability is discussed in terms of the single-center urban population, geographically heterogeneous health practices, and changes in infectious-disease exposure and care patterns before and after lockdowns. |
| Other information | |  | | |
| Funding | 22 | Give the source of funding and the role of the funders for the present study and, if applicable, for the original study on which the present article is based | 14 | Funding sources are listed, including NCI grants, the MSK Cancer Center Support Grant, HHMI, Calico, and NIH support. |

*Give information separately for cases and controls in case-control studies and, if applicable, for exposed and unexposed groups in cohort and cross-sectional studies.

**Note:** An Explanation and Elaboration article discusses each checklist item and gives methodological background and published examples of transparent reporting. The STROBE checklist is best used in conjunction with this article (freely available on the Web sites of PLoS Medicine at http://www.plosmedicine.org/, Annals of Internal Medicine at http://www.annals.org/, and Epidemiology at http://www.epidem.com/). Information on the STROBE Initiative is available at www.strobe-statement.org.
