# Supplementary material for: Deconvolving SARS-CoV-2 mRNA Vaccine Impact on Immunotherapy-Related Survival
Source: Cancer Discov. 2026 Jun 22;16(11):2235–41. doi: 10.1158/2159-8290.CD-26-0403 (PMC13725333; doi:10.1158/2159-8290.CD-26-0403)
Supplement: Supplementary file 8 [file cd-26-0403_supplementary_tables_s1-s2_suppst1.docx]

**Supplemental Tables for “Deconvolving SARS-CoV-2 mRNA vaccine impact on immunotherapy-related survival “**

### **Table S1**. Characteristics of patients treated with immune checkpoint inhibitors (ICI).

N (column-wise category %)

|  |  | | All | Not peri-ICI vaccinated | Peri-ICI vaccinated |
| --- | --- | --- | --- | --- | --- |
| Total, n |  | | 8346 | 6847 | 1499 |
| Cancer type, n (%) | Non-Small Cell Lung Cancer | | 2592 (31.1) | 2177 (31.8) | 415 (27.7) |
|  | Melanoma | | 1016 (12.2) | 866 (12.6) | 150 (10.0) |
|  | Esophagogastric Cancer | | 833 (10.0) | 600 (8.8) | 233 (15.5) |
|  | Bladder Cancer | | 764 (9.2) | 621 (9.1) | 143 (9.5) |
|  | Endometrial Cancer | | 626 (7.5) | 506 (7.4) | 120 (8.0) |
|  | Renal Cell Carcinoma | | 513 (6.1) | 418 (6.1) | 95 (6.3) |
|  | Cancer of Unknown Primary | | 508 (6.1) | 428 (6.3) | 80 (5.3) |
|  | Colorectal Cancer | | 329 (3.9) | 280 (4.1) | 49 (3.3) |
|  | Soft Tissue Sarcoma | | 390 (4.7) | 330 (4.8) | 60 (4.0) |
|  | Breast Cancer | | 385 (4.6) | 304 (4.4) | 81 (5.4) |
|  | Hepatobiliary Cancer | | 390 (4.7) | 317 (4.6) | 73 (4.9) |
| Concurrent cytotoxic chemotherapy | No | | 6202 (74.3) | 5249 (76.7) | 953 (63.6) |
|  | Yes | | 2144 (25.7) | 1598 (23.3) | 546 (36.4) |
| Concurrent CTLA4 blockade | No | | 7344 (88.0) | 6004 (87.7) | 1340 (89.4) |
|  | Yes | | 1002 (12.0) | 843 (12.3) | 159 (10.6) |
| Stage at diagnosis, n (%) | 0 | | 279 (3.3) | 218 (3.2) | 61 (4.1) |
|  | I | | 980 (11.7) | 806 (11.8) | 174 (11.6) |
|  | II | | 1003 (12.0) | 844 (12.3) | 159 (10.6) |
|  | III | | 2298 (27.5) | 1878 (27.4) | 420 (28.0) |
|  | IV | | 3724 (44.6) | 3048 (44.5) | 676 (45.1) |
|  | Unknown | | 62 (0.7) | 53 (0.8) | 9 (0.6) |
| Age at ICI start, mean (SD) | | | 64.5 (12.8) | 64.5 (12.8) | 64.3 (12.9) |
| Sex, n (%) | Female | | 4069 (48.8) | 3316 (48.4) | 753 (50.2) |
|  | Male | | 4277 (51.2) | 3531 (51.6) | 746 (49.8) |
| Race, n (%) | Asian-Far East/Indian Subcont | | 622 (7.5) | 494 (7.2) | 128 (8.5) |
|  | Black or African American | | 534 (6.4) | 434 (6.3) | 100 (6.7) |
|  | Other | | 529 (6.3) | 426 (6.2) | 103 (6.9) |
|  | White | | 6661 (79.8) | 5493 (80.2) | 1168 (77.9) |
| Ethnicity, n (%) | Non-Spanish; Non-Hispanic | | 7490 (89.7) | 6150 (89.8) | 1340 (89.4) |
|  | Spanish/Hispanic/Other | | 856 (10.3) | 697 (10.2) | 159 (10.6) |
| Year of ICI start, n (%) | 2017 | | 1055 (12.6) | 1055 (15.4) | 0 (0.0) |
|  | 2018 | | 1282 (15.4) | 1282 (18.7) | 0 (0.0) |
|  | 2019 | | 1444 (17.3) | 1444 (21.1) | 0 (0.0) |
|  | 2020 | | 1291 (15.5) | 1159 (16.9) | 132 (8.8) |
|  | 2021 | | 1585 (19.0) | 522 (7.6) | 1063 (70.9) |
|  | 2022 | | 1689 (20.2) | 1385 (20.2) | 304 (20.3) |
| ECOG Performance score, n (%) | 0 | | 3389 (40.6) | 2756 (40.3) | 633 (42.2) |
|  | 1 | | 3985 (47.7) | 3282 (47.9) | 703 (46.9) |
|  | 2 | | 451 (5.4) | 379 (5.5) | 72 (4.8) |
|  | 3 | | 59 (0.7) | 53 (0.8) | 6 (0.4) |
|  | 4 | | 7 (0.1) | 7 (0.1) | 0 (0.0) |
|  | Missing | | 455 (5.5) | 370 (5.4) | 85 (5.7) |
| Days from diagnosis to ICI initiation | | mean (SD) | 692.4 (1226.1) | 704.6 (1228.6) | 636.9 (1213.8) |
|  | | Unknown | 63 | 54 | 9 |

### **Table S2.** Characteristics of patients treated with immune checkpoint inhibitors (ICI) stratified by year of ICI start

|  |  | All | Not peri-ICI vaccinated | Peri-ICI vaccinated | p-value^1^ |
| --- | --- | --- | --- | --- | --- |
| **2021** | | | | | |
| Total, n |  | 1585 | 522 | 1063 |  |
| ECOG Performance score, n (%) | 0 | 621 (39.2) | 183 (35.0) | 438 (41.2) | <0.001 |
|  | 1 | 766 (48.3) | 250 (47.8) | 516 (48.5) |  |
|  | 2 | 99 (6.2) | 50 (9.5) | 49 (4.6) |  |
|  | 3 | 10 (0.6) | 6 (1.1) | 4 (0.4) |  |
|  | Missing | 89 (5.7) | 33 (6.5) | 56 (5.3) |  |
| **2022** | | | | | |
| Total, n |  | 1689 | 1385 | 304 |  |
| ECOG Performance score, n (%) | 0 | 713 (42.2) | 576 (41.5) | 137 (45.1) | 0.3 |
|  | 1 | 776 (45.9) | 647 (46.7) | 129 (42.4) |  |
|  | 2 | 72 (4.3) | 54 (3.9) | 18 (5.9) |  |
|  | 3 | 8 (0.5) | 7 (0.5) | 1 (0.3) |  |
|  | Missing | 120 (7.1) | 101 (7.3) | 19 (6.2) |  |

^1^ Fisher’s exact test
